# Soil properties, rather than urbanization time, drive shifts in soil bacterial communities in a subtropical city of China

**DOI:** 10.1128/aem.00958-26

**Published:** 2026-07-10

**Authors:** Yapeng Hao, Qiufang Tan, Liang Kou, Qun Guo, Weimin Wang, Xiangyun Xiong, Hong Liang, Wanyi Zhang, Jing Yang, Jiajia Zheng, Ning Ma, Yu Bai, Shenggong Li

**Affiliations:** 1 Laboratory of Ecosystem Network Observation and Modeling, Institute of Geographic Sciences and Natural Resources Research, Chinese Academy of Sciences 58289, Beijing, China; 2 National Ecosystem Science Data Center, Beijing, China; 3 College of Resources and Environment, University of Chinese Academy of Sciences 74519, Beijing, China; 4 Shenzhen Ecological and Environmental Monitoring Center of Guangdong Province, Shenzhen, China; 5 Guangdong Greater Bay Area, Change and Comprehensive Treatment of Regional Ecology and Environment, National Observation and Research Station, Shenzhen, China; 6 Ecological Quality Comprehensive Monitoring Station of Urban Ecosystem in Shenzhen, Shenzhen, China; 7 Sino-Danish Center for Education and Research, University of Chinese Academy of Sciences 74519, Beijing, China; University of Delaware, Lewes, Delaware, USA

**Keywords:** soil bacteria diversity, urbanized stages, urban land use categories, land use cover change, soil physicochemical properties

## Abstract

Urbanization strongly alters soil environments and may reshape soil bacterial communities (SBC), yet their responses across long-term urbanization stages remain unclear. Using Shenzhen, China, a subtropical city that urbanized within 40 years, we investigated SBC diversity, composition, and co-occurrence network stability across different land-use types and urbanization stages. Soil bacterial α-diversity showed no significant differences between natural forests and urban ecosystems. However, bacterial composition shifted markedly during urbanization, with dominant taxa changing from *Acidobacteriota* and *Verrucomicrobiota* in forests to *Actinobacteriota*, *Chloroflexi*, and *Firmicutes* in urban soils. SBC composition remained strongly associated with soil physicochemical properties rather than converging across urbanization stages, indicating dominant environmental filtering effects. Network analyses further revealed non-linear changes in resistance and resilience stability along the urbanization chronosequence, associated with shifts in network topology and soil properties. Together, our results show that urbanization does not drive a uniform trajectory of SBC assembly; instead, soil environmental conditions under different land-use types primarily shape bacterial composition and potential stability type.

**IMPORTANCE:**

Urbanization is rapidly transforming soil ecosystems worldwide, but its impacts on soil bacterial communities remain poorly understood. We show that urbanization in a subtropical city caused major shifts in dominant bacterial taxa. Urban soils were characterized by a transition from oligotrophic to copiotrophic taxa, accompanied by contrasting changes in network-inferred resistance and resilience stability. These patterns were primarily driven by changes in soil properties associated with land-use change, highlighting the importance of environmental filtering in shaping urban soil microbiomes. Our findings improve understanding of how urbanization restructures underground ecosystems and their potential stability under long-term environmental change.

## INTRODUCTION

Global urbanization is accelerating, and China in particular has witnessed rapid urbanization in the past four decades, driven by its sustained economic growth (1). Urbanization-induced anthropogenic activities (e.g., transportation, industrial practices, and urban greenings) (2–4) continuously alter the soil physicochemical properties (e.g., soil moisture, heavy metal content, pH, and temperature) (5–8), exerting direct or indirect influences on soil microbial communities (9). As integral components of urban soil ecosystems, soil bacteria play a pivotal role in urban ecosystem services—such as nutrient cycling, pollutant degradation, and plant growth—while also influencing urban residents’ health through the activities of pathogenic or beneficial soil bacteria (10, 11). Given their instrumental role in maintaining both urban ecosystem services and human health (12), there is a critical need to deepen our understanding of SBC dynamics during urbanization.

The diversity, composition, and stability of SBC are the keys to be characterized during urbanization (13–15). Based on the ecological niche and community assembly theories, urbanization-induced alterations in SBC may exhibit divergent patterns. On one hand, soil bacterial diversity may increase due to expanded ecological niches resulting from the complex soil environments shaped by the heterogeneity of anthropogenic activities in urban areas (13). On the other hand, reduced diversity and compositional convergence could be observed, driven by niche compression (e.g., plant homogeneity in urban green spaces) (16) and intensified environmental filtering (e.g., selective pressure from high heavy metal concentrations in urban soils) (17). Considering the climate constraint (primarily temperature) on soil microbial activity (18), urbanization-induced shifts in soil bacterial communities are likely to vary with climate zones. For example, studies have reported increased bacterial diversity in urbanized regions of Finland (19), whereas a contrasting trend was observed in Shanghai (10). Thus, there is a critical need to clarify what the specific impacts of urbanization on soil bacteria from different climatic zones are.

Key determinants influencing urban SBC include urbanization stages and urban land use categories (20–22). Soil properties exhibit linear (23) or non-linear (24) variations along urbanization chronosequences. For instance, soil carbon and nitrogen contents, as well as heavy metal concentrations, may show linear increases with advancing urbanization (25, 26), whereas heavy metal accumulation can also exhibit non-linear patterns (24). Such linear or non-linear variation in soil properties substantially influences diverse responses of SBC across different urbanized stages (22). Furthermore, vegetation diversity and variations in structure across land use types (27, 28), combined with regional disparities in the intensity of anthropogenic activities (29), are likely to shape distinct responses in the three aforementioned characteristics of SBC (i.e., diversity, composition, and stability) under urbanization (14, 20). Thus, we need to investigate the successional dynamics of soil bacteria across different urbanization stages and land use types.

Shenzhen serves as a natural laboratory for investigating impacts of rapid urbanization on SBC in subtropical climate zones for two reasons. First, the city experiences a mild subtropical monsoon climate with optimal temperature, light, and water conditions (30), which foster a rich vegetation cover in urban built-up areas (31), resulting in a unique soil bacterial composition and growth patterns that distinguish it from other cities. Second, Shenzhen’s urbanization process has unfolded over a relatively short span of 40 years, during which urban land area has expanded continuously, and land use types have undergone extensive transformation (32). By selecting Shenzhen as a study area and collecting soil samples across different urbanization stages and land use categories, we aimed to elucidate how urbanization stages and land use categories influence the diversity, composition, and stability of SBC. Additionally, this study seeks to uncover the underlying mechanisms governing these dynamics within subtropical urban ecosystems.

## MATERIALS AND METHODS

### Study sites and soil sampling

**Fig 1:**
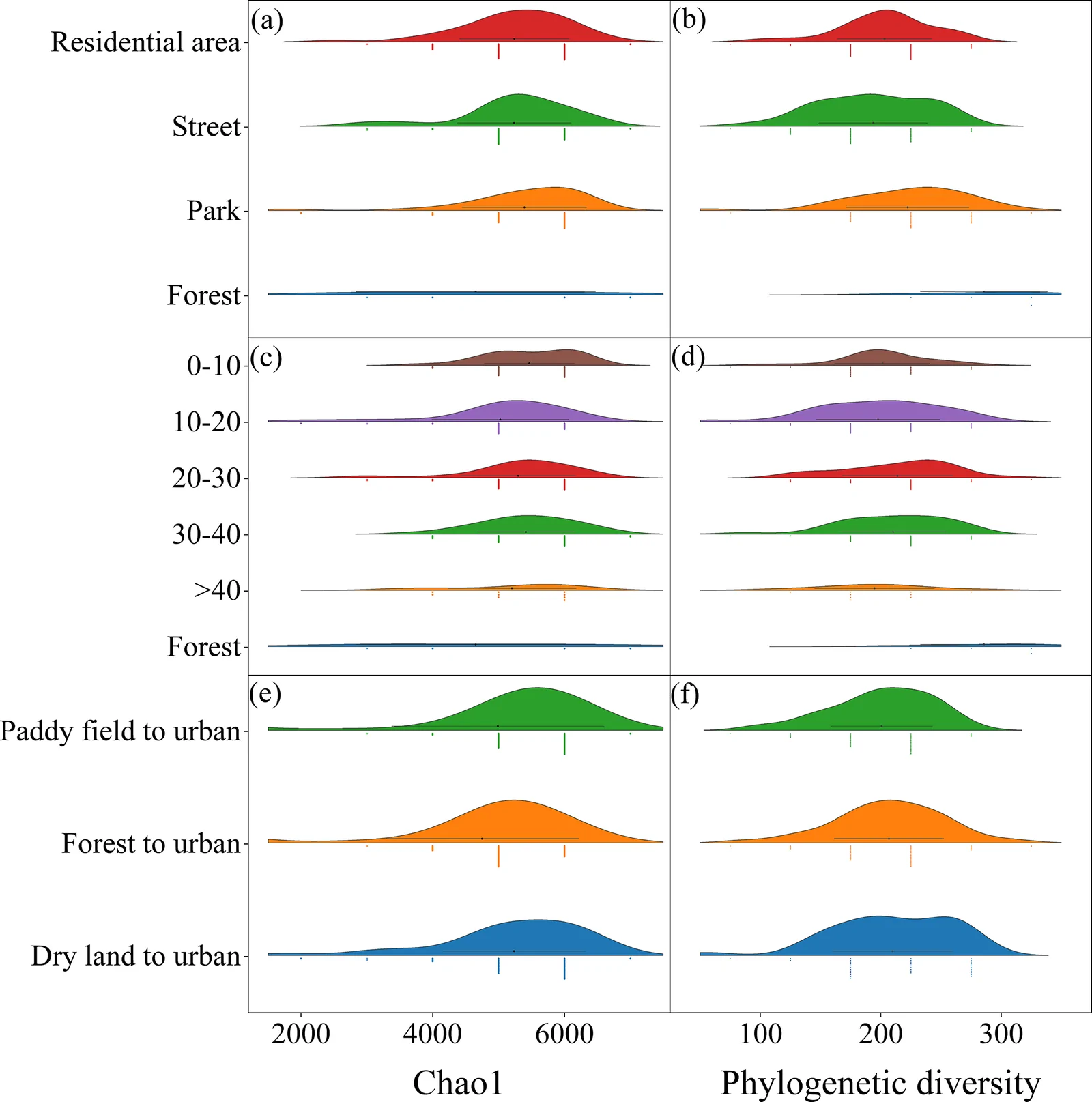
No significant differences in Chao1 and phylogenetic diversity were observed among different urban land use types (**a and b**), various urbanized stages (**c and d**), and original land use types (**e and f**) in Shenzhen City in China.

Shenzhen (113°43′−114°38′E, 22°24′−22°52′N) (Fig. 1) is a coastal megacity located in southeastern China. It has a subtropical monsoon climate with an average annual temperature of ca. 23.3°C and an average annual precipitation of ca. 1,932.9 mm. Shenzhen has undergone a remarkable transformation from a small piscatorial village into a modern metropolis since 1978.

We collected soil samples in July 2023 when the weather was mostly sunny without rain. The sampling sites were in accordance with the land use cover changes from 1980 to 2020 (Fig. S1, China’s Multi-Temporal Land Use and Cover Change Remote Sensing Monitoring Data Set, with a spatio-temporal resolution of 30 m and 10 years) (33; https://www.resdc.cn/DOI/DOI.aspx?DOIID=54). To improve the reliability of site classification, remote-sensing data were further cross-validated using Shenzhen land use records of urban development archives and field observations at the sampling-point scale. Soil sampling was conducted by selecting 3–4 representative soil for each combination of three key forces: urbanization stages (0–10, 10–20, 20–30, 30–40, and >40 years), original land use categories (forest, paddy field, and dry land), and urbanized land use categories (park, street, and residential area (34). Each combination was collected with 3–4 samples, yielding a total of 124 sampling sites across a study area of approximately 2,000 km². At each sampling site, surface soil (0–10 cm depth) was collected by blending five subsamples within a 5 × 5 m plot, placed in sterile bags, and transported to the laboratory in ice boxes immediately. Plant roots were manually excised with sterilized tweezers. Half of the soil samples were immediately frozen at −10°C on the sampling day for subsequent DNA extraction, while the remaining samples were air-dried for analysis of soil properties.

### Molecular analysis of soil bacteria and measurement of soil physicochemical properties

The total DNA of each sample was extracted from 0.25-g soil using the Fast DNA SPIN for Soil (MP Biomedicals, Cambridge, UK). The bacterial 16s rRNA gene was amplified using 338F and 806R primers with Illumina adaptors (35). Each sample triplicate amplification, and electrophoresis was used to verify positive PCR products. Amplicons from triplicate reactions were purified using the AxyPrepDNA Extraction Kit (AXYGEN Company) and mixed in equal density ratios. Sequencing was carried out on an Illumina Miseq platform (2 × 300 bp paired-end reads).

The QIIME2 pipeline (version 2019.10) and the DADA2 plugin with default settings were used to process raw reads (36). Taxonomy was assigned to representative sequences using the SILVA 138.2 database for bacteria (37). AVS (phylotype) abundance tables were normalized to the smallest sample size (42,458) after singletons were excluded. Soil pH was measured using a pH meter. Soil total carbon (TC) and total nitrogen (TN) were determined using an elemental analyzer based on dry combustion. Total phosphorus (TP) was measured following perchloric acid digestion. For other elements (K, Ca, Ti, Cu, and Zn), concentrations were determined using inductively coupled plasma optical emission spectrometry (ICP-OES) after acid digestion. For elements with concentrations below the detection limit of ICP-OES, inductively coupled plasma mass spectrometry (ICP-MS) was used.

### Statistical analysis

The α-diversity of SBC was analyzed by the Chao1 and phylogenetic diversity (PD) indices at the species level (ASV/OTU) for various influences (urban land use types, urbanized stages, and original land use types). The Chao1 is a widely used and reliable indicator in the analysis of diversity (38). Considering the evolutionary history of species, PD index provides a more informative assessment of community diversity compared with richness (39). We use the least significant difference (LSD) method to analyze the averaged differences in α-diversity among land use types or urbanization chronosequences. To further characterize within-group variability in α-diversity, we calculated the mean, standard deviation (SD), and coefficient of variation (CV; SD/mean) for each group, and assessed homogeneity of variances using the Fligner–Killeen test. The Spearman’s rank correlation was used to analyze the correlations among α-diversity of SBC (calculated at the species level), soil physicochemical properties, and different affecting forces, with *P*-values adjusted using the Benjamini and Hochberg false discovery rate (FDR) method.

Partial least squares discriminant analysis (PLS-DA) was used to identify differences in species composition at the species level affected by each factor. PLS-DA is a supervised multivariate method that can highlight compositional differences among predefined groups and has been widely applied in omics and microbial community analyses (40, 41). To ensure that the observed patterns were not dependent on the supervised method, we also performed non-metric multidimensional scaling (NMDS) based on Bray–Curtis dissimilarity. The NMDS ordination showed relatively weak separation among groups, whereas PLS-DA highlighted group differences more clearly. The NMDS results are provided in the Supplementary Materials (Fig. S2). Permutational multivariate analysis of variance (PERMANOVA) was employed to identify significant differences in species composition among different urbanization stages or land use types. Spearman’s rank correlation was employed to assess the correlations among the composition of SBC (calculated at the phylum level) and soil physicochemical properties. LDA effect size (LEfSe) at the species level was determined to identify the significant and abundant taxa affected by each factor (42). Significance was set at *P* < 0.05, and taxa with LDA >4.5 were reported.

Though lines of research provided various methods to study bacterial stability (43, 44), recent findings suggested that topological analyses of soil bacterial co-occurrence networks may offer novel insights (45) into bacterial interactions (46) and community stability (47). Co-occurrence networks with their topological features have been widely used to investigate co-occurrence patterns among species and community stability in complex environments (48).

In this study, co-occurrence networks were constructed at the species level. Spearman’s method was used for correlation analysis, with *P*-values adjusted using the FDR method. The cutoff of adjusted *P*-value was 0.01. Deconvolution method was used to discern the direct correlation dependencies, and the random matrix theory-based method was used to determine the cutoff of the modified coefficients. The coefficient cutoff was 0.70. Before co-occurrence network analysis, species with relative abundances lower than 0.001 were excluded. The topological features of the co-occurrence network were analyzed and reported, including nodes number, edge number, positive correlation proportion, average degree, average shortest path length, edges density, and modularity.

To assess the robustness of network topology, a resampling (bootstrap) approach was applied by randomly selecting 80% of samples within each group and reconstructing the networks for 10 iterations. The corresponding topological metrics, e.g., modularity, average degree, and edge density, were calculated for each iteration and visualized using violin plots. Differences among groups were further evaluated using one-way analysis of variance (ANOVA), followed by least significant difference (LSD) tests. To evaluate the effect of abundance thresholds on network structure, an additional set of networks was constructed using a stricter cutoff (relative abundance >0.01). Differences in topological features between the two thresholds (0.001 vs 0.01) were assessed using independent *t*-tests. Furthermore, for networks constructed with the 0.001 threshold, node topological roles were classified based on within-module connectivity (Zi) and among-module connectivity (Pi). Nodes were categorized into peripheral nodes, connectors, module hubs, and network hubs using threshold values of Zi = 2.5 and Pi = 0.62 (49). Microbial communities with high modularity areoften positively associated with higher network-inferred resistance stability (RT [50–52]), but lower resilience stability (RL) (53, 54), while communities with higher average degree and edge density generally demonstrate stronger network-inferred RL (55–57).

All statistical analyses except LEfSe analysis were performed with R version 4.1.1 (58). Chao1 index and PD index were calculated by the package “vegan.” The LSD test was calculated by the “LSD.test” function in the package “agricolae.” Spearman’s rank correlation analysis was performed with the “correlate” function in the package “linkET.” PLS-DA was calculated by the “plsda” function in the package “mixOmics.” PERMANOVA was performed with the “adonis2” function in the package “pairwiseAdonis.” The co-occurrence network and topological features were performed with the package “igraph.” LEfSe analysis was carried out according to the instructions on the website (https://www.bioincloud.tech). Network visualization was generated with Gephi (v. 0.10.1). After importing the node and edge data into Gephi, the layout was set to “Fruchterman Reingold” with an area of 10,000, gravity of 10.0, and speed of 1.0. By clicking “Run,” the positions of the nodes and edges were continuously adjusted until a stable network structure was generated.

## RESULTS

### Soil physicochemical properties

Soil physicochemical properties varied significantly among land use types and urbanization stages (Fig. S3 to S5). Across land use types, TP was significantly higher in residential areas (0.09% ± 0.14%) than in parks (0.04% ± 0.03%) and forests (0.02% ± 0.01%, *P* < 0.05), while the C:N ratio was higher in streets (13.47% ± 1.79%) and parks (13.23% ± 1.77%) compared with residential areas (12.25% ± 1.92%). The N:P ratio was highest in forests (11.08% ± 5.31%, *P* < 0.05), whereas soil pH was lowest in forests (6.28 ± 0.37, *P* < 0.05). In addition, soil Cu content was highest in residential areas (0.004% ± 0.009%, *P* < 0.05, Fig. S3). Along the urbanization gradient, TP peaked at 10–20 years (0.06% ± 0.04%) after urbanization, while the C:N ratio decreased significantly with increasing urbanization age (*P* < 0.05). In contrast, N:P ratio, K content, and pH exhibited unimodal patterns, with peaks at 20–30 (5.66% ± 2.75%), 30–40 (1.74% ± 0.41%), and 20–40 years (7.04 ± 0.28, 7.07 ± 0.34), respectively (*P* < 0.05). Soil Cu content showed a declining trend after urbanization, with a rapid decrease during the first 10 years (0.004% ± 0.011%), although this trend was not statistically significant (Fig. S4). No significant differences in soil properties were detected among different original land use types after urbanization (*P* > 0.05, Fig. S5).

### Effects of land use categories and urbanized stages on α-diversity of SBC

There were no significant differences in α-diversity (in terms of Chao1 and phylogenetic diversity [PD]) of SBC between natural ecosystems (forests) and urban land use categories (parks, streets, and residential areas) or among the three urban land use categories (*P >* 0.05, Fig. 1a and 1b) in Shenzhen City. However, variability differed among land use types. In particular, forests tended to have lower bacterial α-diversity and exhibited relatively high within-group variability, e.g., higher CV, which may obscure potential differences in α-diversity (Table S1).

The α-diversity of SBC was also not associated with the duration of the natural lands that have been urbanized (*P >* 0.05, Fig. 1c and 1d). The Fligner–Killeen test further indicated that variance did not differ significantly among urbanization stages, suggesting comparable levels of dispersion and a low likelihood that unequal variance contributed to the non-significant results (Table S1). Additionally, the original land use types had no significant effect on Chao1 or PD of SBC after urbanization (*P >* 0.05, Fig. 1e and 1f). Although the Fligner–Killeen test indicated significant differences in variance among groups (*P* < 0.05), the coefficients of variation were relatively similar across these groups (Table S1). Correlation analysis of α-diversity of SBC with environmental factors showed that soil properties, land use categories, urbanization history, and original land use categories had no significant effect on either Chao1 index or PD (*P* > 0.05, Table S2).

### Effects of land use categories and urbanized stages on species composition of SBC

**Fig 2:**
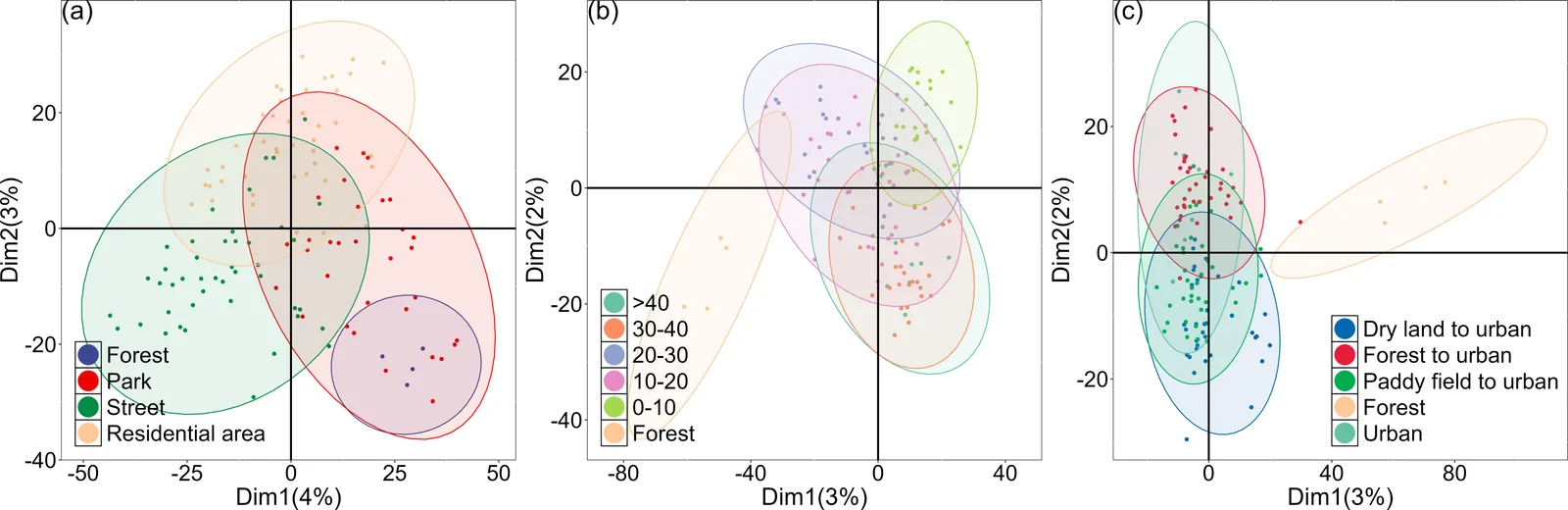
Comparison of the soil bacterial species composition among different present (**a**) or original (**c**) land use types and urbanized stages (**b**) in Shenzhen City in China.

Significant differences in species composition (SC) of SBC were observed among forests, streets, residential areas, and parks (*P <* 0.05, Fig. 2a and Table S2), implying shifts of SC after urbanization and variations among different land use types of urban areas. Along with the time of urbanization, SC showed stage-specific patterns. In particular, samples within the 10-year urbanization stage showed a distinct SC compared with other stages (*P <* 0.05, Fig. 2b and Table S3). In contrast, no significant differences were detected among 20-, 40-, and over 40-year stages (*P >* 0.05, Fig. 2b and Table S3). However, the SC of the 30-year group showed a significant difference from that after 30 years, implying that community composition may still vary during intermediate stages of urban development. Additionally, SC did not significantly link to what kind of land use types they originally were (*P >* 0.05, Fig. 2c and Table S4).

**Fig 3:**
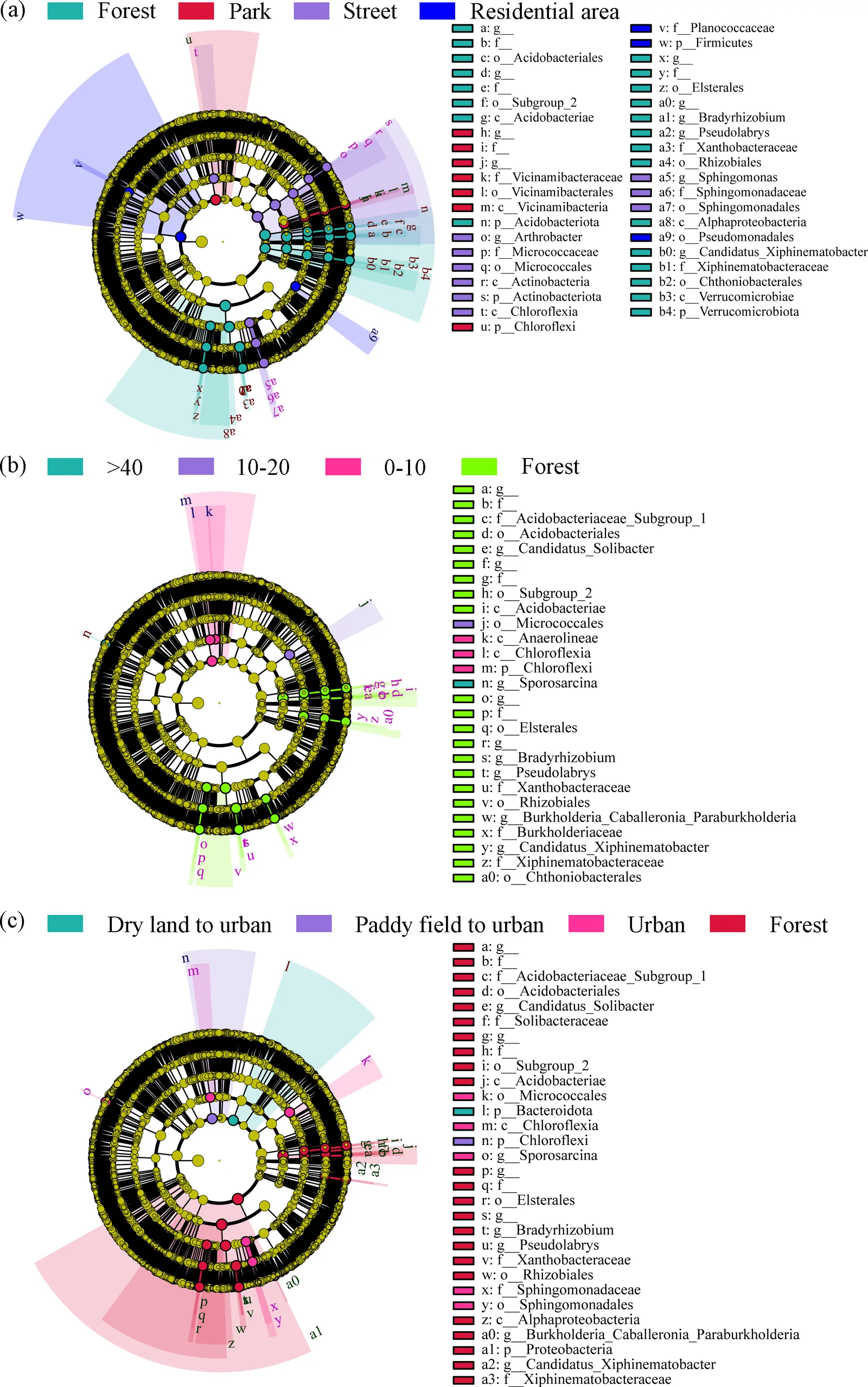
Strict version of LEfSe results on microbial communities in Shenzhen City in China. The cladogram indicates the taxa (highlighted with small circles and shading) showing different abundance values (according to LEfSe) in different land-use categories (**a**), urbanized stages (**b**), or original land-use categories (**c**). For each taxon (circle), the color denotes the significantly higher abundance of the taxon in the corresponding group. p_, phylum; c_, class; o_, order; f_, family.

LEfSe analysis revealed differences in dominant soil bacterial taxa among forests and present urban land-use categories (Fig. 3a). Forest soils were dominated by taxa of p_*Acidobacteriota* and p_*Verrucomicrobiota* (*P* < 0.05, Fig. 3a). Street and park soils were all composed of higher abundances of taxa p_*Actinobacteriota* and p_*Chloroflexi* (*P* < 0.05, Fig. 3a). For various urbanization stages, p_*Chloroflexi*, o_*Micrococcales* (an order from p_*Actinobacteria*), and g_*Sprosarcina* dominate the soil bacteria in soils with less than 10, 10–20, and over 40 years of urbanization, respectively (*P* < 0.05, Fig. 3b and Fig. S6b). From the perspective of original urbanization land-use categories, p_*Bacteriodota* were dominant in soil where the original type of land was dry land, whereas p_*Chloroflexi* were more abundant in soil where it was originally paddy field (*P* < 0.05, Fig. 3c), implying the original land use types may exert certain effects on the dominant bacterial species, though no effects on biodiversity or SC.

The study highlighted key differences in SBC composition across seven dominant phyla: *Acidobacteriota*, *Actinobacteriota*, *Bacteroidota*, *Chloroflexi*, *Firmicutes*, *Proteobacteria*, and *Verrucomicrobiota*, among which almost all bacteria phyla were positive to N/*P* (except *Firmicutes* and *Chloroflexi*). Other nutrient-related indicators were observed, i.e., TP, K, TN, and TC. Positive correlations were observed between TP and *Firmicutes*, and between K and *Actinobacteriota*. Negative correlations were observed for TN and TC with *Chloroflexi*, and TP with *Verrucomicrobiota*, *Bacteroidota*, and *Acidobacteriota*. Additionally, soil pH and heavy metal elements, e.g., Ca, Zn, Cu, and Ti, also affected dominant species. Soil pH, Ca, Zn, and Ti positively correlated with *Bacteroidota*, *Actinobacteriota*, and *Verrucomicrobiota*, respectively. Whereas negative correlations were found between soil pH and Ti and *Firmicutes. Acidobacteriota* and *Verrucomicrobiota* were also negatively related to Ca, Cu, and Zn.

### Topological features of the soil bacterial co-occurrence network

**Fig 4:**
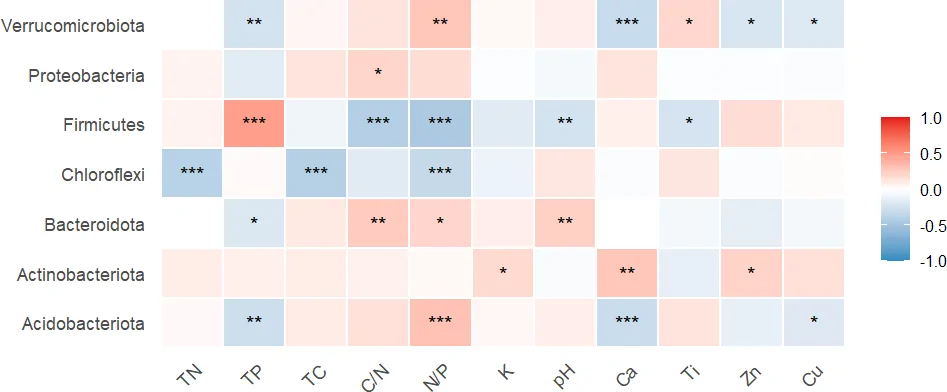
Spearman correlations between soil physicochemical properties and the abundances of bacterial phylum. *, *P* < 0.05, **, *P* < 0.01, ***, *P* < 0.001.

**Fig 5:**
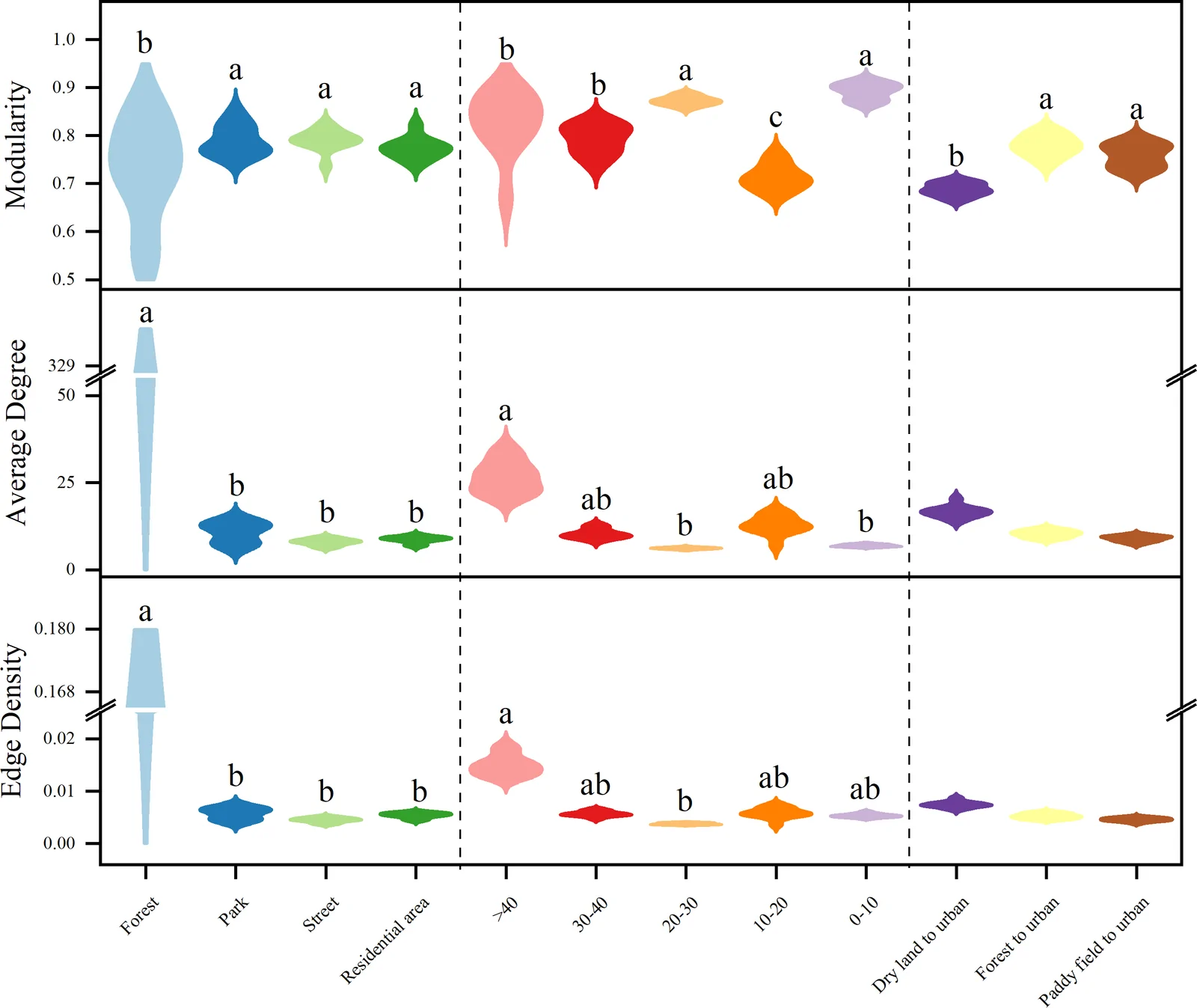
Topological features of the soil bacterial co-occurrence network based on ASVs with relative abundance greater than 0.001.

The modularity (MD) of the co-occurrence network with relative abundance greater than 0.001 in urban land was higher than that in forests (*P* < 0.05, Fig. 5), implying a potential increase in network modular structure following urbanization, while the average degree (AD) and edge density (ED) in urban land were lower than that in natural forest (*P* < 0.05, Fig. S7a through d), indicating a denser pattern of microbial associations in forest soils. Within the framework of network topology, MD has often been associated with resistance-related stability (RT), whereas AD and ED have been interpreted as indicators potentially linked to resilience-related properties (RL). Along the urbanization chronosequences, network topological features exhibited stage-dependent patterns (Fig. 5 and Fig. S7e through i). During the early stage of urbanization (0–10 years), MD reached relatively high levels (*P* < 0.05), while AD (*P* < 0.05) and ED remained comparatively low, suggesting a more modular but less connected network structure. As urbanization progressed, MD declined and reached a minimum at 10–20 years, followed by a subsequent increase, whereas AD and ED exhibited the opposite pattern, peaking at the intermediate stage (20–30 years) and decreasing thereafter. Such non-linear dynamics were not strictly consistent with urbanization stages but were more likely associated with shifts in soil properties. In particular, potassium (K) showed significant variation across stages (Fig. S4) and was positively correlated with the relative abundance of *Actinobacteriota* (Fig. 4). Zi–Pi analysis further revealed that *Actinobacteriota* tended to occupy more central roles (e.g., module hubs and connectors) in urban-associated networks compared with forest soils (Fig. S8e through i), suggesting their potential contribution to maintaining network connectivity.

Moreover, network topology also varied among original land use types (Fig. 5), indicating that legacy effects of land conversion may additionally shape microbial network organization. Notably, although networks constructed using a relative abundance threshold of >0.01 exhibited significantly different absolute topological values (*P* < 0.05, Table S6), the overall trends across urbanization stages remained consistent, supporting the robustness of these patterns (Fig. S9 and Table S7).

## DISCUSSION

### Neither urbanization nor land use types had significant effects on α-diversity of bacteria community

There was no significant effect of urbanization (present or original land use types and various urbanization stages) on soil bacterial α-diversity in this study. Although α-diversity tended to be lower in forest soils compared with urban land-use types, these differences were not statistically significant, indicating that urbanization did not impose a consistent directional change in bacterial diversity. Reports have shown that urbanization (e.g., residential areas, parks, transportation areas, sports fields, and urban farms) significantly increased (13, 19, 59–61) or decreased (62) soil bacterial abundance/diversity, while others indicate no significant effect of urbanization on soil bacterial abundance/diversity (12, 63–66). These controversial results may result from the intensified and complicated anthropogenic activities in urban ecosystems. Contrasting to the natural ecosystems, there are multiple land management measures including fertilization and irrigation, locally adapted landscaping, such as increased vegetation cover and planting of indigenous species, and urbanized constructions for industrial production and transportation. All of these activities significantly and intricately impacted the soil properties, including pH (19), nutrient availability, organic matter (61), humidity (67), temperature (11), particle size (61), and heavy metal content (68, 69), leading to an increase or decrease in soil bacterial diversity. For one thing, anthropogenic greening actions, such as planting, fertilization, tilling, and irrigation, can enhance soil organic matter by stimulating above-ground biomass, which will foster bacterial growth and thereby potentially increase soil bacterial diversity (13, 70). However, the generally low species diversity in urban greening often results in a homogeneous composition of soil organic matter, which does not promote the diversity of soil nutrients and thus fails to enhance soil bacterial diversity (59, 71). On another hand, fertilization can act as a strong environmental filtering factor through severe soil acidification, leading to the decreased soil bacterial diversity according to the community assembly theory (72). Additionally, heavy metal contamination is one of the main changes in the soil environment following urbanization (73), while its effects on soil bacterial diversity are contingent. For instance, the urbanization-induced increase in the diversity heavy metal elements can act as a new stimulus to the reassembly of soil bacteria, thereby increasing their diversity due to the varying sensitivity of bacterial species to different heavy metal elements (14). However, excessive concentrations of soil heavy metals (e.g., Cr and Cd) may reduce soil bacterial diversity by inhibiting the growth of most of bacteria, which in turn strengthens environmental filtering (14, 17).

The sampling sites in this study (street, residential areas, and parks) were all subjected to complex urban management practices and were covered with greenery that has a relatively homogeneous species composition (e.g. roadside greenbelt consists solely of *Ficus microcarpa L. f.*). Meanwhile, an increasing trend in the concentrations of some heavy metals (e.g., Cu and Zn) was observed at these sampling sites. These urbanization-induced environmental changes exerted both positive and negative effects on bacterial diversity. Given that minor changes in the soil environment can lead to substantial shifts in the structure of SBC (74), the coexistence of these opposing effects results in no significant impact of urbanization on bacterial diversity in this study.

### Both urbanization stages and land use types had significant effects on bacteria composition at the phylum level

Although urbanization had no significant impact on the α-diversity of SBC, our study revealed that the composition of SBC in this subtropical metropolis was significantly altered by urbanization. In natural ecosystems, soil bacteria were dominated by *Acidobacteriota* and *Verrucomicrobiota*, whereas urban soils were enriched with key bacterial phyla, such as *Actinobacteriota*, *Chloroflexi,* and *Firmicutes*. This shift may be attributed to urbanization-induced changes in the soil environment. In accordance with previous studies, our analysis showed that *Acidobacteriota* and *Verrucomicrobiota* were negatively correlated with soil TP, Cu, and pH (22, 75), while soil TP, Cu, and acidity were lower in Shenzhen’s forest soils (Fig. S3), fostering a favorable soil environment for the proliferation of *Acidobacteriota* and *Verrucomicrobiota*. With the progression of urbanization, significant decreases were observed in soil TN, TC, and N/*P* ratios, accompanied by increases in soil pH, Zn, and Cu (Fig. S4). As reported in the literature and confirmed in our study, *Chloroflexi* showed a negative correlation with soil TN, TC, and N/*P* ratios, while both *Chloroflexi* and *Firmicutes* were positively correlated with Zn and Cu (76). Additionally, all three dominant bacterial phyla (*Actinobacteriota*, *Chloroflexi*, and *Firmicutes*) were positively correlated with soil pH (75, 77). These collective changes in soil properties provided ideal conditions for the growth of *Actinobacteriota*, *Chloroflexi*, and *Firmicutes* in urbanized areas.

Moreover, the SC of SBC exhibited non-linear changes along the urbanization gradient. Within the first 10 years of urbanization, during which soil TN, TC, and N/P ratios showed a significant decline compared with those in natural ecosystems (Fig. S4), *Chloroflexi* became the dominant bacteria in urban soil. Notably, a pronounced shift occurred at 20–30 years of urbanization, where microbial community composition differed significantly from both earlier and later stages. At this stage, no single dominant phylum was observed. This pattern likely reflects a threshold in soil environmental conditions, as multiple soil properties, e.g., N/P, TP, and TC, underwent directional shifts during this period (Fig. S4). Such coordinated changes in soil chemistry may alter environmental filtering, leading to a transient reorganization of microbial communities and the breakdown of dominance patterns. The aforementioned variations in soil properties were closely related to anthropogenic activities in urban areas. First, activities that increased soil TP in urban areas included the use of phosphorus-containing building materials, the application of phosphorus fertilizers in urban green spaces, and the discharge of industrial wastewater (78, 79). Second, activities that decreased soil TN and TC in urban areas included mechanical removal of surface soil, soil compaction, and vegetation management practices, e.g., the removal of grass clippings, leaves, and other organic debris (79). Finally, factors contributing to the accumulation of heavy metals, e.g., Zn and Cu, and increases in soil pH in urban areas included industrial production, dense traffic, and infrastructure construction, along with the ash emissions and dust generated by these activities (25, 80).

### Both urbanization stages and land use types affected the co-occurrence network stability of the soil bacteria

Network-derived topological properties suggested that urbanization substantially altered the organization of soil bacterial co-occurrence networks. Compared with natural forests, urban soils exhibited higher MD but lower AD and ED, indicating a shift from densely connected microbial associations toward more compartmentalized network structures. Forest soils generally provide relatively stable environmental conditions and continuous organic resource inputs (19, 81), which may promote stronger microbial interconnections and support greater RL-related properties following disturbance. In contrast, urban soils are characterized by greater environmental heterogeneity and anthropogenic disturbance (82, 13, 83), potentially weakening network connectivity while favoring the relatively independent modules, consistent with previous studies (84). The non-linear variation in network topology across urbanization stages further suggests that microbial network organization was shaped more strongly by soil physicochemical conditions than by urbanization time itself. In particular, K variation was closely associated with network restructuring. K content varied significantly among urbanization stages and was positively correlated with the relative abundance of *Actinobacteriota* (Fig. 4 and Fig. S4), which occupied more central topological roles in urban-associated networks. Given their broad metabolic capacity and strong environmental adaptability (85, 86), *Actinobacteriota* may contribute disproportionately to maintaining microbial interactions under disturbed urban conditions. These findings suggest that environmental filtering plays an important role in shaping microbial network organization during urbanization.

## Data Availability

The data set supporting the conclusions of this article is available in the NCBI SRA repository under BioProject no. PRJNA1437393. The bacterial species composition data can be found at https://github.com/YP-Hao/Paper_data-The-impact-of-urbanization-on-soil-bacteria.

## SUPPLEMENTAL MATERIAL

Supplemental material aem.00958-26-s0001.docx Fig. S1 to S9; Tables S1 to S7.

ASM does not own the copyrights to Supplemental Material that may be linked to, or accessed through, an article. The authors have granted ASM a non-exclusive, world-wide license to publish the Supplemental Material files. Please contact the corresponding author directly for reuse.
