# Supplementary material for: Soil properties, rather than urbanization time, drive shifts in soil bacterial communities in a subtropical city of China
Source: Appl Environ Microbiol. 2026 Jul 10;92(8):e00958-26. doi: 10.1128/aem.00958-26 (PMC13488327; doi:10.1128/aem.00958-26)
Supplement: Supplementary file 1 [file aem.00958-26-s0001.docx]

Figure S1. The land use cover change and distribution of sampling sites across the Shenzhen City.

Figure S2. NMDS ordination of soil bacterial community composition based on Bray–Curtis dissimilarity, comparing samples among different present (a) and original (c) land-use types and urbanization stages (b) in Shenzhen, China. Ellipses represent 95% confidence intervals.

Figure S3. Boxplots showing the distribution of different soil properties in samples associated with different urban land-use categories. Different letters above the bars indicate significant difference between treatment (*P* <0.05).

Figure S4. Line graphs showing the distribution of different soil properties in samples associated with different urbanized stages. Different letters above the bars indicate significant difference between treatment (*P* <0.05).

Figure S5. Boxplots showing the distribution of different soil properties in samples associated with urban have been converted from different original land use types. Different letters above the bars indicate significant difference between treatment (*P* <0.05).

Figure S6. Histogram of the LDA scores calculated for taxa differently abundant in different urban land-use categories (a), urbanized stages (b) or original land use types (c).

Figure S7. The stability analyzed by the co-occurrence network in Shenzhen City of China. (a) Park, (b) Street, (c) Residential area, (d) Forest, (e) >40, (f) 30-40, (g) 20-30, (h) 10-20, (i) 0-10, (j) Dry land to Urban, (k) Forest to Urban, (l) Paddy field to Urban. Co-occurrence networks were constructed based on OTUs with relative abundances >0.001, using significant Spearman correlations (r < −0.7 or r > 0.7) with an adjusted *P*-value < 0.01. The sizes of the nodes were scaled to their betweenness centrality scores to indicate their relative importance in each module.

Figure S8. Zi–Pi plots showing the topological roles of nodes across different networks. Nodes represent ASVs, and *Actinobacteriota* are highlighted in red. The plot is divided into four regions based on within-module connectivity (Zi = 2.5) and among-module connectivity (Pi = 0.62): peripheral nodes (Zi ≤ 2.5, Pi ≤ 0.62), connectors (Zi ≤ 2.5, Pi > 0.62), module hubs (Zi > 2.5, Pi ≤ 0.62), and network hubs (Zi > 2.5, Pi > 0.62). Background colors indicate these topological roles.

Figure S9. Topological features of the soil bacterial co-occurrence network based on ASVs with relative abundance greater than 0.01.

Table S1. Results of Fligner–Killeen tests for homogeneity of variances and coefficients of variation (CV) of α-diversity indices (Chao1 and phylogenetic diversity, PD) across different grouping factors.

| Group factor | Group | Chao1  (mean ± SD) | CV  (Chao1) | Fligner–Killeen  *P*-value (Chao1) | PD  (mean ± SD) | CV  (PD) | Fligner–Killeen  *P*-value (PD) |
| --- | --- | --- | --- | --- | --- | --- | --- |
| Present land-use types | Forest | 4651.77±1818.69 | 39.1% | 0.037 | 330.85±96.44 | 29.2% | 0.138 |
|  | Park | 5391.04±940.92 | 17.5% |  | 374.79±52.26 | 13.9% |  |
|  | Street | 5234.66±864.10 | 16.5% |  | 373.83±52.91 | 14.2% |  |
|  | Residential area | 5240.04±831.00 | 15.9% |  | 372.164±46.41 | 12.5% |  |
| Urbanized stages | 0-10 | 5464.98±685.83 | 12.5% | 0.129 | 384.81±36.33 | 9.4% | 0.197 |
|  | 10-20 | 5026.28±1037.66 | 20.6% |  | 355.50±58.73 | 16.5% |  |
|  | 20-30 | 5295.87±869.23 | 16.4% |  | 373.55±52.39 | 14.0% |  |
|  | 30-40 | 5413.92±743.48 | 13.7% |  | 385.42±42.60 | 11.1% |  |
|  | >40 | 5202.35±974.10 | 18.7% |  | 369.80±53.15 | 14.4% |  |
| Original land-use types | Dry land to urban | 5234.26±1080.31 | 20.6% | 0.011 | 373.11±63.63 | 17.1% | 0.034 |
|  | Forest to urban | 5162.60±730.29 | 14.1% |  | 361.84±41.51 | 11.5% |  |
|  | Paddy field to urban | 5452.651 | 13.8% |  | 386.32±41.01 | 10.6% |  |

Table S2. Spearman’s Rank Correlation Analysis between soil properties, land-use categories, urbanized stages, original land-use categories, and $\alpha$-diversity of SBC in Shenzhen City of China.

|  |  | Chao1 index | | phylogenetic diversity | |
| --- | --- | --- | --- | --- | --- |
|  |  | coefficients | *P* value | coefficients | *P* value |
| Soil properties | TN | -0.14 | 0.37 | -0.16 | 0.24 |
|  | TP | -0.07 | 0.75 | -0.02 | 0.95 |
|  | TC | -0.08 | 0.64 | -0.13 | 0.41 |
|  | C/N | 0.02 | 0.95 | -0.02 | 0.95 |
|  | N/P | -0.02 | 0.95 | -0.09 | 0.59 |
|  | K | 0.02 | 0.95 | 0.04 | 0.86 |
|  | pH | 0.04 | 0.87 | 0.08 | 0.64 |
|  | Zn | -0.08 | 0.64 | -0.05 | 0.82 |
|  | Cu | -0.05 | 0.82 | 0.00 | 0.99 |
| Present land-use types | Forest | -0.05 | 0.79 | -0.08 | 0.68 |
|  | Park | 0.13 | 0.42 | 0.06 | 0.78 |
|  | Street | -0.04 | 0.88 | 0.02 | 0.95 |
|  | Residential area | -0.06 | 0.78 | -0.04 | 0.83 |
| Urbanized stages | 0-10 | 0.09 | 0.62 | 0.09 | 0.60 |
|  | 10-20 | -0.14 | 0.34 | -0.19 | 0.16 |
|  | 20-30 | 0.03 | 0.91 | 0.02 | 0.95 |
|  | 30-40 | 0.06 | 0.78 | 0.11 | 0.49 |
|  | >40 | 0.00 | 0.99 | 0.02 | 0.95 |
| Original land-use types | Dry land to urban | 0.02 | 0.95 | 0.06 | 0.78 |
|  | Forest to urban | 0.15 | 0.31 | 0.20 | 0.13 |
|  | Paddy field to urban | -0.15 | 0.33 | -0.23 | 0.05 |

The different groups of urbanization factors were transformed to dummy variables (Presence, 1; Absence, 0). *, *P* < 0.05.

Table S3. Differences in bacterial species composition among different urban land-use categories.

| **type** | **Df** | **F** | **p** |
| --- | --- | --- | --- |

| Park vs Street | 1 | 4.65 | **0.001** |
| --- | --- | --- | --- |
| Park vs Residential area | 1 | 3.95 | **0.001** |
| Park vs Forest | 1 | 4.00 | **0.002** |
| Street vs Residential area | 1 | 2.21 | **0.002** |
| Street vs Forest | 1 | 5.22 | **0.001** |
| Residential area vs Forest | 1 | 5.05 | **0.001** |

Table S4. Differences in bacterial species composition among different urbanized stages

| **type** | **Df** | **F** | **p** |
| --- | --- | --- | --- |

| >40 vs 30-40 | 1 | 0.91 | 0.538 |
| --- | --- | --- | --- |
| >40 vs 20-30 | 1 | 1.55 | **0.05** |
| >40 vs 10-20 | 1 | 1.05 | 0.333 |
| >40 vs 0-10 | 1 | 1.53 | **0.038** |
| 30-40 vs 20-30 | 1 | 1.74 | **0.011** |
| 30-40 vs 10-20 | 1 | 1.20 | 0.183 |
| 30-40 vs 0-10 | 1 | 1.62 | **0.016** |
| 20-30 vs 10-20 | 1 | 0.92 | 0.562 |
| 20-30 vs 0-10 | 1 | 2.20 | **0.001** |
| 10-20 vs 0-10 | 1 | 1.80 | **0.012** |
| Forest vs >40 | 1 | 3.89 | **0.002** |
| Forest vs 30-40 | 1 | 4.67 | **0.001** |
| Forest vs 20-30 | 1 | 4.12 | **0.001** |
| Forest vs 10-20 | 1 | 3.63 | **0.001** |
| Forest vs 0-10 | 1 | 6.06 | **0.001** |

Table S5. Differences in bacterial species composition among different original land use types

| **type** | **Df** | **F** | **p** |
| --- | --- | --- | --- |

| Dry land to urban vs Paddy field to urban | 1 | 0.92 | 0.566 |
| --- | --- | --- | --- |
| Dry land to urban vs Forest to urban | 1 | 1.04 | 0.384 |
| Paddy field to urban vs Forest to urban | 1 | 1.11 | 0.335 |
| Forest vs Dry land to urban | 1 | 4.01 | **0.003** |
| Forest vs Forest to urban | 1 | 4.89 | **0.003** |
| Forest vs Paddy field to urban | 1 | 4.57 | **0.003** |
| Urban vs Dry land to urban | 1 | 1.18 | 0.334 |
| Urban vs Paddy field to urban | 1 | 1.12 | 0.335 |
| Urban vs Forest to urban | 1 | 1.10 | 0.335 |
| Urban vs Forest | 1 | 3.89 | **0.005** |

Table S6 Comparison of topological features of co-occurrence networks constructed using different relative abundance thresholds (0.01 vs 0.001), with significance assessed by t-tests.

| Group | average degree  *P*-value | edges density  *P*-value | modularity  *P*-value |
| --- | --- | --- | --- |
| Park | **0.00** | **0.00** | 0.30 |
| Street | **0.00** | **0.00** | 0.15 |
| Residential area | **0.00** | **0.00** | 0.31 |
| Forest | **0.00** | 0.60 | 0.12 |
| >40 | **0.00** | **0.00** | **0.03** |
| 30-40 | **0.00** | **0.00** | **0.05** |
| 20-30 | **0.00** | **0.00** | **0.00** |
| 10-20 | **0.00** | **0.00** | 0.31 |
| 0-10 | **0.00** | **0.00** | **0.00** |
| Dry land to urban | **0.00** | **0.00** | **0.00** |
| Forest to urban | **0.00** | **0.00** | **0.00** |
| Paddy field to urban | **0.00** | **0.00** | 0.20 |

Table S7 The topological features of the co-occurrence network

| Group | relative abundances >0.01 | | | relative abundances >0.001 | | |
| --- | --- | --- | --- | --- | --- | --- |
|  | average degree  (mean±SD) | edges density  (mean±SD) | modularity  (mean±SD) | average degree  (mean±SD) | edges density  (mean±SD) | modularity  (mean±SD) |
| Park | 3.21±0.19 | 0.0316±0.0020 | 0.78±0.03 | 10.76±2.98 | 0.0058±0.0012 | 0.79±0.03 |
| Street | 2.48±0.20 | 0.0305±0.0034 | 0.81±0.04 | 7.94±0.96 | 0.0045±0.0005 | 0.79±0.02 |
| Residential area | 2.62±0.25 | 0.0349±0.0050 | 0.76±0.04 | 8.66±0.98 | 0.0054±0.0005 | 0.77±0.02 |
| Forest | 19.98±7.77 | 0.1278±0.0455 | 0.67±0.06 | 210.92±72.95 | 0.1176±0.0407 | 0.73±0.11 |
| >40 | 2.67±0.84 | 0.0489±0.0190 | 0.76±0.09 | 26.64±4.55 | 0.0149±0.0020 | 0.83±0.07 |
| 30-40 | 2.28±0.33 | 0.0304±0.0031 | 0.82±0.04 | 10.30±1.43 | 0.0057±0.0005 | 0.79±0.03 |
| 20-30 | 2.58±0.29 | 0.0353±0.0034 | 0.77±0.04 | 6.15±0.28 | 0.0038±0.0002 | 0.87±0.01 |
| 10-20 | 4.53±0.33 | 0.0377±0.0030 | 0.70±0.04 | 12.80±2.71 | 0.0058±0.0010 | 0.71±0.03 |
| 0-10 | 1.56±0.25 | 0.0485±0.0119 | 0.83±0.05 | 6.90±0.35 | 0.0053±0.0004 | 0.89±0.02 |
| Dry land to urban | 2.96±0.25 | 0.0300±0.0022 | 0.81±0.03 | 16.64±1.72 | 0.0074±0.0006 | 0.69±0.01 |
| Forest to urban | 3.89±0.39 | 0.0430±0.0049 | 0.69±0.03 | 10.30±1.07 | 0.0053±0.0005 | 0.78±0.02 |
| Paddy field to urban | 2.81±0.32 | 0.0308±0.0047 | 0.77±0.04 | 8.95±0.87 | 0.0046±0.0004 | 0.76±0.02 |
